# Health Service Utilization for Mental, Behavioural and Emotional Problems among Conflict-Affected Population in Georgia: A Cross-Sectional Study

**DOI:** 10.1371/journal.pone.0122673

**Published:** 2015-04-08

**Authors:** Ivdity Chikovani, Nino Makhashvili, George Gotsadze, Vikram Patel, Martin McKee, Maia Uchaneishvili, Natia Rukhadze, Bayard Roberts

**Affiliations:** 1 Curatio International Foundation, Tbilisi, Georgia; 2 Global Initiative on Psychiatry, Tbilisi, Georgia and Ilia State University, Tbilisi, Georgia; 3 London School of Hygiene and Tropical Medicine, London, United Kingdom; Merced, UNITED STATES

## Abstract

**Background:**

There is large gap in mental illness treatment globally and potentially especially so in war-affected populations. The study aim was to examine health care utilization patterns for mental, behavioural and emotional problems among the war-affected adult population in the Republic of Georgia.

**Methods:**

A cross-sectional household survey was conducted among 3600 adults affected by 1990s and 2008 armed conflicts in Georgia. Service use was measured for the last 12 months for any mental, emotional or behavioural problems. TSQ, PHQ-9 and GAD-7 were used to measure current symptoms of PTSD, depression and anxiety. Descriptive and regression analyses were used.

**Results:**

Respondents were predominantly female (65.0%), 35.8% were unemployed, and 56.0% covered by the government insurance scheme. From the total sample, 30.5% had symptoms of at least one current mental disorder. Among them, 39.0% sought care for mental problems, while 33.1% expressed facing barriers to accessing care and so did not seek care. General practitioners (29%) and neurologists (26%) were consulted by the majority of those with a current mental disorder who accessed services, while use of psychiatric services was far more limited. Pharmacotherapy was the predominant type of care (90%). Female gender (OR 1.50, 95% CI: 1.25, 1.80), middle-age (OR 1.83, 95% CI: 1.48, 2.26) and older-age (OR 1.62, 95% CI: 1.19, 2.21), possession of the state insurance coverage (OR 1.55, 95% CI: 1.30, 1.86), current PTSD symptoms (OR 1.56, 95% CI: 1.29, 1.90) and depression (OR 2.12, 95% CI: 1.70, 2.65) were associated with higher rates of health service utilization, while employed were less likely to use services (OR 0.71, 95% CI: 0.55, 0.89).

**Conclusions:**

Reducing financial access barriers and increasing awareness and access to local care required to help reduce the burden of mental disorders among conflict-affected persons in Georgia.

## Introduction

Health system responses to mental illness are almost always inadequate, with a large gap between those who require mental health care and those who actually receive effective treatment[1] [2],[3]. The gap is potentially also wide in war-affected communities, with their high mental disease burden from the trauma and daily stressors of war and their weak health infrastructure [4],[5],[6]. Yet the limited research on utilization of health services by persons exposed to armed conflict has largely been among those seeking asylum abroad or among military veterans, with much less being undertaken among the much higher numbers of civilians that are still living in conflict or post-conflict settings.

Georgia has around 200,000 internally displaced persons (IDPs). The majority were displaced by the separatist wars of the 1990s in South Ossetia and Abkhazia, and around 20,000 also remain displaced from the 2008 war with Russia over South Ossetia [7],[8]. Approximately 40% of the IDPs reside in collective centers, with the remaining IDPs living in private accommodation. Collective centers represent former public buildings such as kindergartens, schools, administrative buildings or newly built “cottages” in a purpose-built villages constructed by the government after the 2008 conflict. IDP communities are characterised by poor living conditions, high unemployment, poverty, limited integration with local communities and financial barriers to access health care and medicines [9],[10],[11].

Services for mental disorders are funded by the government through the *State Program for Mental Health* (SPMH). The SPMH offers services to all citizens of Georgia, with services delivered in outpatient clinics (‘psycho-neurological dispensaries’) and specialised inpatient facilities or psychiatric departments in general hospitals [12]. Outpatient and inpatient care is free, including medications. Inpatient services cover all mental disorders, while some disorders (e.g. anxiety and obsessive-compulsive disorders) are excluded from the outpatient package (see Table 1).

**Table 1:** Mental health services by different state programs and providers.

Health care provider	Health care facility	Funding Source	MH services
Pharmacy	Retail drug store	Out of pocket payment	Drug selling; advice on drugs
GP	GP office, ambulatory, policlinic	Government insurance	Management of mild depression and prescription of antidepressants, free outpatient MH. However, free medications are not provided.
Neurologist	Policlinic	Government insurance	No mental health disorders are covered by Government insurance, except management of mild depression and prescription of antidepressants (but free MH medications are not provided)
Neurologist	Hospital	Government insurance,	No mental health disorders are covered by Government insurance, except management of mild depression and prescription of antidepressants (but free MH medications are not provided).
Psychiatrist	Outpatient clinic (dispensary)	SPMH	Outpatient care (defined list of mental disorders), counseling, free outpatient MH medication provision
Psychiatrist	General hospital or psychiatric hospital	SPMH	Inpatient care (all mental disorders requiring inpatient treatment), counseling, free medications
Psychiatrist, Psychologist, Psychotherapist	Private clinic	Out of pocket payment	Counseling, psychotherapy, medication therapy
Psychiatrist, Psychotherapist, Social worker	Psychosocial rehabilitation centers; Mobile services	Donor funds; Few centers funded by SPMH	Multidisciplinary case management, free outpatient medication provision

SPMH, State Program for Mental Health.

In addition to the SPMH mental health care, GPs also provide care for mild depression management under the government’s general insurance scheme. GPs are also authorised to prescribe antidepressant medications but the costs of these medications are not covered. Since 2006, the general health insurance scheme is free for persons living below the poverty line [13] and those who were displaced from the 2008 conflict and who remained in collective centers [14]. However, those displaced during the conflict in the early 1990s or in the 2008 conflict and who have subsequently returned to their villages are only included if they meet the eligibility criteria for poverty applied to the general population.

There is extremely limited research on the mental health of IDPs in Georgia despite risk-factors of trauma exposure, forced displacement, daily stressors and impoverishment [15]. To the best of our knowledge, no quantitative study has previously been conducted on mental health service utilisation among conflict-affected persons in Georgia. The only study on this topic we could identify was a qualitative study conducted in 2012 among 39 older IDPs investigated health care seeking behaviour for mental heath problems [16]. Indeed, there has been no quantitative research on mental health service utilisation patterns among the general population in Georgia.

This paper presents findings from the first large-scale epidemiological mental health study with the adult conflict-affected adult population in Georgia (for further details, please see [15] [17] [18]). The aim of this paper is to examine health care utilization patterns for mental, behavioural and emotional problems among the war-affected adult population in the Republic of Georgia. The specific objectives are to: 1) measure health service utilization rates; 2) identify reasons why those in need did not seek care; 3) describe types of health services used; and 4) identify determinants of health service utilization.

## Methods

### Sample

The study used a cross-sectional survey design with multi-stage random sampling, with stratification by region and displacement status. A total sample of 3600 men and women aged 18 years and over was determined for the overall study and not specifically for the heath service utilization component [15]. The sample consisted of 1200 respondents from each of the 3 main conflict-affected populations in Georgia: those displaced as a result of conflicts in the 1990s (‘1990s IDPs’); those displaced after the 2008 conflict (‘2008 IDPs’); and individuals affected by 2008 conflict who have returned to their home areas after being displaced or who did not change their location but experienced armed conflict (‘returnees’).

Primary sampling units (N = 360; 120 per population group) were selected based on probability proportional to size using a sampling frame of population lists in formal and informal IDP settlements provided by the Ministry of Internally Displaced Persons, and lists of villages in the border region with South Ossetia provided by the Governor’s office in Shida Kartli region. These were considered to be the most accurate lists available. The IDP lists were complete as they were recently updated by the Ministry of Internally Displaced Persons. Likewise the returnee’s lists were complete and accurate as they were based on regular information provided by administrative heads of villages. Within each primary sampling unit, the random walk method was used to randomly select households. Within the selected household, one person (aged ≥18 years) was randomly selected to be interviewed (based on nearest birthday). If the person was not reached after 3 visits (on different days and at different times), the next household on the route was visited, with the same process used for refusals or interrupted interviews to reach the desired sample of 3600 respondents. The overall response rate for the survey was 79%. Non-responses were due to: household member still not available after 3 call backs (N = 800); refusals (N = 166); and interrupted interviews (N = 10).

Data collection took place between October and December 2011. Face-to-face interviews took place in the respondents’ homes and the questionnaires were administered by trained fieldworkers. All interviews were conducted in Georgian. All respondents provided written informed consent prior to their inclusion in the study. Full respondent anonymity was assured. People with severe intellectual or mental impairment with reduced ability to communicate were excluded from the study. The National Council on Bioethics in Georgia and the London School of Hygiene and Tropical Medicine provided ethical approval for this study.

### Measure of health service utilization

Respondents were asked whether they had feelings such as anxiety, nervousness, depression, insomnia or any other emotional or behavioural problems for which they sought health care during the 12 months prior to interview. Those that had sought some kind of care were then asked what type it was. These were classified as: pharmacy; General Practitioner’s (GP) office, ambulatory or policlinic; neurologist at policlinic; neurologist or therapist at hospital; psychiatrist at outpatient clinic (dispensary); psychiatrist at hospital; psychosocial center, private mental health specialist; outreach/mobile services. These are described in Table 1. Respondents who had sought care were also asked what type of treatment they received, classified as: medication treatment, counselling and psychotherapy/psychosocial support. The terms “counselling” and “psychotherapy/psychosocial support” were not specifically explained, as they are commonly understood. In general the terms are commonly defined as: counselling—a conversation with a doctor where the doctor gives advice, prescribes drugs, etc; and psychotherapy—treatment without medications through interactions with a specialist (psychologist, psychiatrist). Respondents who self-reported having mental, emotional or behavioural problems but did not use health services were asked additional questions about reasons for not seeking care.

### Measurement of mental disorders:

In addition to the single question on self-reported mental, emotional or behavioural problems, instruments were used to measure the prevalence of symptoms of Post-Traumatic Stress Disorder (PTSD), depression and anxiety. PTSD was measured using the Trauma Screening Questionnaire (TSQ) which consists of 10 questions on experiencing PTSD symptoms over the past 1 week with yes = 1, no = 0 responses. The overall score (sum) ranges from 0 to 10 with TSQ’s suggested cut-off of >6 used to indicate current PTSD symptoms that may be indication of possible PTSD [19]. Symptoms of depression were measured using the Patient Health Questionnaire (PHQ-9), consisting of 9 questions about experience of symptoms of depression over the last 2 weeks. The responses ranged from 0 = not at all to 3 = nearly every day. The item scores are summed to produce a total score range 0 to 27 with the PHQ-9’s suggested cut-off of ≥10 used to indicate current depression symptoms or possible depression disorder [20]. Symptoms of anxiety were measured using the Generalised Anxiety Disorder (GAD-7) instrument, which consists of 7 questions on experience of anxiety symptoms over the last 2 weeks. The GAD-7 questionnaire uses the response options and scoring as PHQ-9, with suggested cut-off ≥10 to indicate moderated current anxiety symptoms or possible anxiety disorder [21]. TSQ, PHQ-9, and GAD-7 showed good reliability with Cronbach’s alpha scores of 0.86, 0.86, 0.90 respectively; and results from a separate test-retest mini survey (N = 110) produced intraclass correlation coefficients (ICC) of 0.97, 0.98, and 0.96 respectively. Further details on the reliability and validity of the study instruments are described by Makhashvili at al [15]. There was overlap between the 3 measures and we did not treat them separately as mutually exclusive conditions. The confidence intervals for them in the results are therefore intended to show the precision of the results rather than for a comparison between the mental health conditions.

Socio-demographic characteristics were also included in the questionnaire. These included age, gender, education level, marital status, living conditions, employment status, household economic status.

Instrument translation used standard procedures involving: (i) translation from English into Georgian using professional translators, with translations reviewed by Georgian mental health experts individually and then as a group for cultural relevance, content and concept consistency, clarity and understanding; (ii) a back-translation to check for accuracy, consistency and equivalence, with adjustments made accordingly; and (iii) piloting and field testing to refine the instruments further [22]. Trained and experienced professional fieldworkers were used, with the interviewers trained by the research staff with participation of the mental health expert.

### Statistical analyses

Patterns of service utilization by type of mental health disorder and by type of services used, and reasons for not using services, were described using Chi square tests to compare groups. To assess the influence of different variables on health service utilization, multivariate logistic regression was carried out. The dependent variable—service utilization—is defined as visiting any type health care provider at formal health services for behavioural or emotional problem during last 12 months. Health care provider refers to pharmacist, GP, neurologist, mental health specialist (psychiatrist, psychologist, psychotherapist). Health services refer to pharmacy, GP office / policlinic, general hospital, psychiatric outpatient clinic, psychiatric hospital/department, private clinic, psychosocial rehabilitation center, mobile services. In the first stage of the regression analysis two blocks of independent variables were formed of socio-demographic and health related variables. The socio-demographic block comprised gender, age, marital status, education, economic status, employment, displacement status and possession of health insurance. A health related block included current PTSD, depression and anxiety symptoms as one subgroup and co-morbidity (> 1 of these possible mental disorders) as a separate variable. Multivariate regression analysis was run separately for each block and co-morbidity was independently tested by univariate analyses. The independent variables that were not significantly (P<.05) associated with the dependent variable were excluded from the final model. Multicollinearity diagnostics was conducted for: for socio-demographic variables (gender, age, economic status, employment, displacement status, possession of health insurance) with separate outcomes of possible PTSD, depression, anxiety and comorbidity. The findings indicated no significant multicollinearity.

The sample was weighted to reflect the actual proportions of 'old IDPs', 'new IDPs' and 'returnees' in the overall conflict-affected population of Georgia. Cases for which there were missing data were dropped from the analysis (<2% for the key dependent and independent variables of interest). Data analysis was performed in SPSS 18.0. Statistical significance was taken as P<0.05.

## Results

The sample characteristics are presented in Table 2. Overall 65% were women, most were married, and 69.6% had complete secondary education, and 56% were covered by the government’s general insurance scheme (94.6% among new IDPs, 59.2% among old IDPs, and 40.9% of among returnees). There were no major differences in the socio-demographic characteristics among the 3 IDP groups. Turning to mental health, 23.5% of respondents were classified with current PTSD symptoms, 14.4% with current depression symptoms and 10.9% with current anxiety symptoms. In the study sample, 30.5% had symptoms of at least one disorder, while 12.7% had symptoms of more than one disorder and 5.6% had symptoms of all three disorders. Further details on the prevalence of mental disorder symptoms by displacement groups are reported elsewhere [15].

**Table 2:** Description of the sample.

	**N = 3600**	**%**
**Gender**		
Male	1259	35.0
Female	2341	65.0
**Age**		
18–39	1248	34.7
40–59	1254	34.8
60+	1098	30.5
**Marital status**		
Single	617	17.2
Married/Cohabitating	2123	59.0
Seperated/DivorcedWidowed	855	23.8
**Education**		
Completed higher	760	21.1
Completed secondary school	2506	69.6
Primary/incomplete secondary	334	9.3
**Economic status** *		
Very good/Good/ average	1651	45.9
Bad/ Very bad	1947	54.1
**Employment**		
Unemployed	1288	35.8
Employed	829	23.0
Housewife /on maternity leave	448	12.4
Retired due to age or disability	963	26.8
Student	71	2.0
**Health Insurance**		
Government insurance scheme	2017	56.0
Private or corporate insurance	77	2.1
No insurance	1486	41.3
**Displacement status**		
New IDPs	335	9.3
Old IDPs	2053	57.0
Returnees	1211	33.7
**Mental disorder symptoms**		
PTSD symptoms ^1^	844	23.5
Depression symptoms ^2^	519	14.4
Anxiety symptoms ^3^	394	10.9
No symptoms of mental disorder	2503	69.5
Symptoms of at least one disorder	1096	30.5
Symptoms for more than one disorder	458	12.7
Symptoms for all three disorders	203	5.6

^1^ = TSQ score >6.

^2^ = PHQ-9 score of ≥10.

^3^ = GAD-7 score of ≥10.

* self-reported against these categories.

### Service utilization

Table 3 shows that a quarter (24.8%) of all respondents self-reported mental, behavioural or emotional problems and sought formal care during the preceding 12-month period. However, it is informative to focus on those meeting the criteria for having current symptoms of the three mental disorders of PTSD, depression and anxiety. Thirty nine percent of those with current symptoms of any of these 3 disorders (i.e. ≥ 1 disorder) and reporting having mental, emotional or behavioural problems over the past 12 months sought care; 33.1% reported such problems but did not seek care, and 27.4% did not report problems or seek care. Almost half of those meeting the criteria for current depression symptoms (48.1%), or when more than one disorder was present (47.5%), reported problem and sought care. A third of those with current symptoms for any of the three mental disorders reported emotional and behavioural problems but did not seek care. The proportion is similar among those with symptoms of PTSD or anxiety and having more than one condition.

**Table 3:** Service utilization for mental health, any emotional or behavioural problems during last 12 months by presence of mental health disorder symptoms.

	**Total**	**Self-reported problem and sought care**			**Self-reported problems but did not seek care**			**Did not have self-reported problem to seek care for**		
		n	%	95% CI	N	%	95% CI	n	%	95% CI
Total sample	N = 3600	892	24.8	23.4–26.2	706	54.8	53.2–56.4	1971	19.6	18.3–20.9
Any mental health disorder	N = 1096	427	39	35.7–42.3	363	33.1	30.0–36.4	300	27.4	24.4–30.5
Comorbidity	N = 458	217	47.5	42.9–52.6	157	34.4	29.5-39-4	79	17.2	13.6–21.5
PTSD symptoms ^1^	N = 844	335	39.7	36.4–43.0	274	27.7	24.7–30.7	234	32.5	29.3–35.6
Depression symptoms ^2^	N = 519	250	48.1	43.8–52.4	176	16.7	13.4–19.9	86	34	29.9–38.1
Anxiety symptoms ^3^	N = 394	168	42.7	37.7–47.6	137	21.6	17.6–25.7	85	34.9	30.1–39.6

The Confidence Intervals (CI) are provided to show the precision of the results and are not intended for a comparison between the mental health groupings as there is overlap between the mental health grouping and they were not treated as mutually exclusive.

^1^ = TSQ score >6.

^2^ = PHQ-9 score of ≥10.

^3^ = GAD-7 score of ≥10.

**Fig 1:**
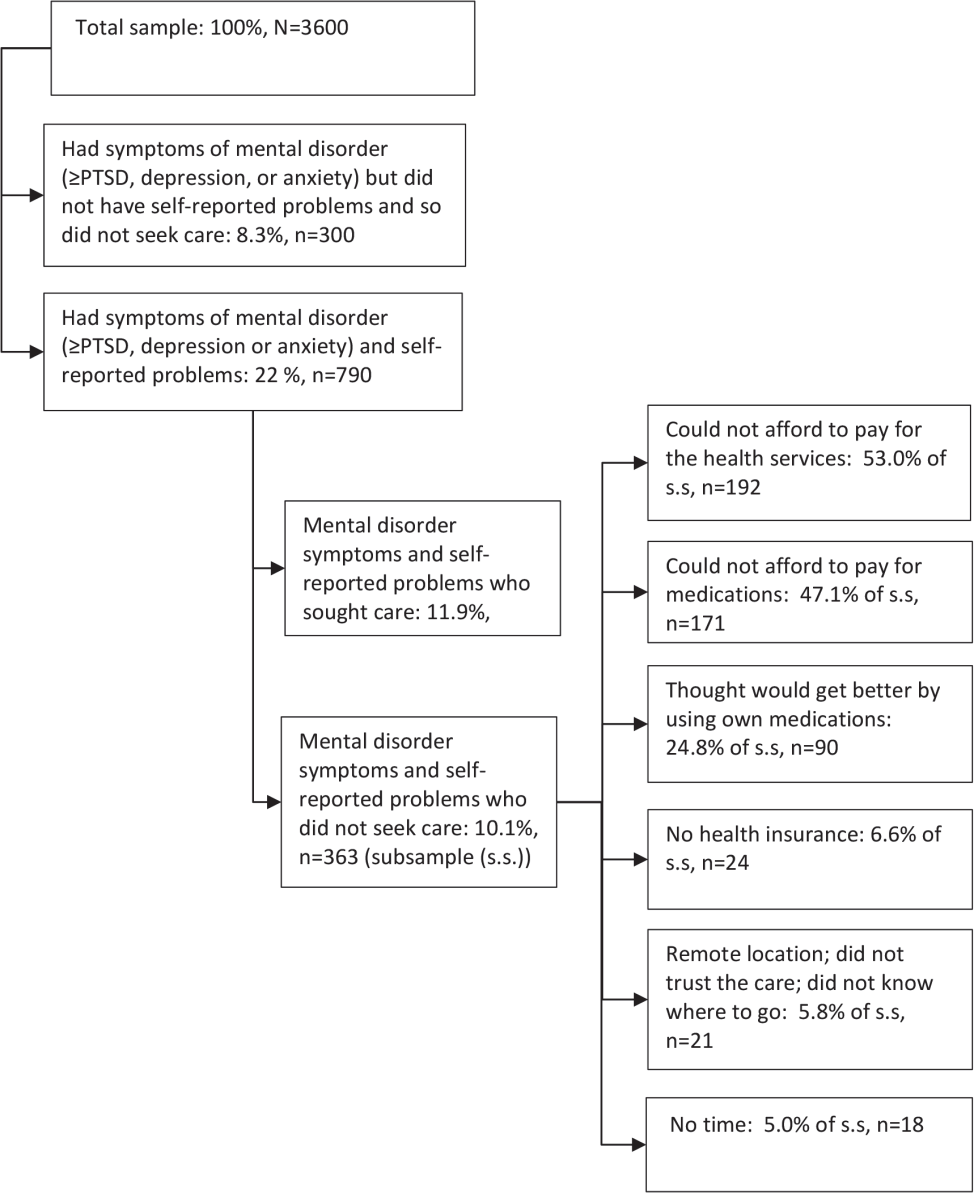
Reasons of not seeking health care in the presence of mental health symptoms and any emotional or behavioural problems (multiple answers allowed).

From our total study sample, 790 (22%) individuals screened for current mental disorder symptoms and self-reported emotional or behavioural problem during last 12 months. Of these, 363 individuals did not seek care. The reasons why they did not seek care are shown in Fig 1 (multiple responses were possible). The most common reasons were inability to afford care or drugs, with very few not seeking treatment because they either did not know where to go or had no insurance.

### Types of services utilized

Table 4 presents the service providers and types of care used by those individuals who sought health care due to current emotional and behavioural problems, separating those not having current symptoms of one of the mental disorders measured in the study (i.e. PTSD, depression or anxiety) from those with symptoms of at least one of these disorders. Overall, there were not statistically significant differences in utilization rates between the two groups except for those with no current mental health disorder symptoms making statistically significant lower use of GP/ambulatory/ policlinic services (39.8% *vs*. 46.6% p = 0.04), psychiatric dispensary services (0.6% *vs*. 2.3% p = 0.035) and medication treatment (81.5% vs. 90.2% p<0.001). The majority (around 70%) in both groups used pharmacies, and around half in both groups consulted neurologist at hospital or outpatient clinic. Very few (1.2%) with current mental disorder symptoms attended psychiatric hospital during last 12 months. A further analysis (data not shown) found no statistically significant (P<.05) difference in the pattern of use among those screened with symptoms of the different mental health disorders.

**Table 4:** Type of care used among individuals who contacted formal health services for any mental health, emotional or behavioural problems during last 12 months by presence of current mental disorder symptoms.

	**No current mental disorder symptoms**		Any current mental disorder symptoms ^1^		Total	
	N = 465		N = 427		N = 892	
Type of service provider	%	95% CI	%	95% CI		
Pharmacy	**72.3**	68.2–76.4	**69.1**	64.7–73.5	**70.7**	67.7–73.7
Only pharmacy use	**17.0**	13.7–20.5	**13.8**	10.6–17.2	**15.6**	13.2–18.0
GP office /ambulatory / policlinic	**39.8**	35.4–43.2	**46.6**	44.2–51.3	**43.1**	39.8–46.3
Only GP use	**29.0**	24.5–33.9	**28.6**	23.9–33.6	**28.8**	24.1–33.7
Therapist/ Neurologist at Hospital	**34.2**	29.8–38.4	**30.2**	25.9–34.7	**32.3**	29.2–35.4
Neurologist at polyclinic	**20.5**	16.8–24.2	**26.0**	21.8–30.2	**23.1**	20.4–25.9
Outreach/mobile services	**4.5**	2.6–6.3	**7.0**	4.7–9.5		
Psychiatric dispensary	**0.6**	0.1–1.1	**2.3**	1.2–3.9	**1.5**	0.7–2.3
Psychosocial center, Private MH specialist	**1.9**	0.6–3.1	**2.3**	0.9–3.9	**2.1**	1.2–3.1
Psychiatric hospital	**0.6**	0.1–1.2	**1.2**	0.1–2.1	**0.8**	0.2–1.4
Type of care						
Medication treatment	**81.5**	78.0–85.1	**90.2**	87.2–92.9	**85.6**	83.3–87.9
Counselling	**84.1**	80.8–87.5	**84.5**	81.2–88.1	**84.4**	82.0–86.8
Psychotheraphy/ psychosocial support	**2.8**	1.3–4.2	**4.9**	2.9–7.0	**3.8**	2.5–5.0

^1^ = Screened with symptoms of one of more of possible PTSD (TSQ score >6), depression (PHQ-9 score of ≥10), anxiety (GAD-7 score of ≥10.

Insured individuals were more likely to consult GPs for emotional and behavioural problems than those without health insurance (45.8% and 37.3% respectively, p = 0.019), while those insured were less likely to use only a pharmacy than those without insurance (13.8% and 19.8% respectively, p = 0.025) (not shown in the table).

The most common type of care was medication treatment followed by counselling, while very few received psychotherapy or psychosocial support (Table 4). No significant difference was found in the type of care used between respondents with different mental disorder symptoms.

### Characteristics associated with health care utilization

The multivariate regression analysis shows that displacement status (old, new and returnee) and economic condition were not associated with the probability of using services. However, being female (OR 1.50. 95% CI: 1.25, 1.80), being in middle age (OR 1.83 95% CI: 1.48, 2.26) and 40 years and older (OR 1.62 95% CI: 1.19, 2.21) and having the government’s general insurance coverage (OR 1.55, 95% CI: 1.30, 1.86) are significantly associated with higher rates of health service utilization for emotional and behavioural problems (Table 5). Those who were employed were less likely to use services (OR 0.71, 95%CI 0.55–0.89). Being screened with current symptoms of PTSD (OR 1.56, 95% CI 1.29–1.90) or depression (OR 2.12, 95%CI 1.70–2.65) significantly increased odds of service use but anxiety symptoms did not in the univariate analysis and so anxiety was not included in the final model. Respondents with symptoms of more than one of the three disorders were more likely to consult health services.

**Table 5:** Correlates of service utilization, multivariate logistic regression, final model.

	Service Utilization					
	**n**	**%**	**Odds Ratio**		**95% CI**	
**Gender**						
Male	240	19.1	ref			
Female	652	27.9	**1.50**	**	**1.25**	**1.80**
**Age**						
18–39	187	15.0	ref			
40–59	343	27.4	**1.83**	**	**1.48**	**2.26**
60+	361	32.9	**1.62**	**	**1.19**	**2.21**
**Economic status**						
Very good/Good/ average	313	19.0	ref			
Bad/ Very bad	577	29.6	1.19		0.99	1.42
**Employment**						
Unemployed	298	23.1	ref			
Employed	139	16.8	**.71**	*	**.55**	**.89**
Housewife /on maternity leave	106	23.7	.84		.64	1.11
Retired due to age or disability	343	35.6	1.16		.87	1.56
**Displacement status**						
Returnee	257	21.2	ref			
New IDP	92	27.5	.93		.70	1.24
Old IDP	542	26.4	.84		.62	1.15
**Health Insurance**						
No insurance	268	18.0	ref			
Private or corporate insurance	18	23.7	1.44		.82	2.53
Government scheme	602	29.8	**1.55**	**	**1.30**	**1.86**
**PTSD** ^**1**^						
No current disorder symptoms	556	20.2	ref			
Current disorder symptoms	335	39.7	**1.56**	**	**1.29**	**1.90**
**Depression** ^**2**^						
No current disorder	642	20.8	ref			
Current Disorder	250	48.2	**2.12**	**	**1.70**	**2.65**
**Co-morbidity** ^**3**^ ***						
Symptoms of one or no current disorder	675	21.5	ref			
Symptoms of more than one disorder	217	47.4	**2.29**	**	**1.85**	**2.84**

Separate regression model run for (1) socio-demographic variables and current PTSD and current depression symptoms; (2) socio-demographic variables and comorbidity. The results for socio-demographic variables, PTSD symptoms and depression symptoms are shown from the first model. There were no statistically significant difference in the results of socio-demographic variables between the first and the second model.

^1^ = TSQ score >6.

^2^ = PHQ-9 score of ≥10.

^3^ = GAD-7 score of ≥10.

* p < 0.05.

** p < 0.01.

*** Co-morbidity is current symptoms more than 1 disorder of PTSD, depression and anxiety.

## Discussion

This study provides new information on patterns of use of health services among those with assessed current mental disorder symptoms among conflict-affected persons in Georgia. No significant difference in service use among the different categories of IDPs and returnees which we henceforth collectively refer to as the war-affected population.

We found that only just over a third of those with a current mental disorder sought any assistance from health services. The remainder (61%) did not use services because they did not report the presence of problems, despite meeting criteria for a current mental health disorder (27.4%) or faced real or perceived barriers to accessing care (33.1%).

This study adds to a sparse existing literature on this topic among conflict-affected civilian populations in low and middle income countries, most of which has been conducted in the Balkans. A study conducted 8 years after the war in Kosovo found that 72% of people had used medical services in the past 12 months [23]. Another study from Kosovo, among female civilians 10 years after the war, found that more than half used health care services during the previous three months but only small minority used specialised mental health services [24]. A study of war-affected populations from the Balkan region observed general service use rates of between 61% to 94% and psychiatric service use ranged between 1.9% to 20.9% [6]. The other study among traumatised population from war-affected Balkan countries examined service use from the beginning of the conflict among individuals with mental disorders. Twenty six percent of those with current PTSD used mental health services, as did 18.1% of those with other mental disorders [25]. A study conducted using a similar methodology in Croatia found that 38.8% of individuals with current PTSD utilized mental health services since the beginning of the war [26]. However, comparison with these studies is challenging due to different study time periods and methodologies.

Our study findings on the factors influencing service utilization are consistent with existing evidence. Being female and middle or old age (40 and up) were significantly associated with service use. Higher utilization by women is a consistent finding in studies among war-affected populations [6], [23], [27]. Those who are employed were less likely to use health services for mental or behavioural problems but previous research finds an inconsistent association of employment and service use; one study of a war-affected population in Kosovo showed higher rates of utilization among employed persons [23] but another, of individuals with severe mental illness, found that steady employment was associated with significantly lower outpatient use [28]. Among individuals with current symptoms of mental health disorders, depressive disorder and PTSD symptoms were associated with higher odds of services use. Increased likelihood of service use of individuals with depressive disorder was also reported by previous studies [2],[27]. Our findings with regard to PTSD also resonate with other research among war affected population [6],[29],[25],[30],[31] and among civilian population [32]. As expected, co-existence of more than one disorder was associated with increased use of health services [33].

To put our results into a broader context, estimates of a treatment gap (i.e. the proportion of individuals who require mental health care but do not receive treatment) among non-conflict-affected persons in the WHO Europe region vary from 45% for people with Major Depression to 62% for people with Generalized Anxiety Disorder [34]. More globally, treatment gaps for serious cases of mental disorders are estimated at around 35–50% in developed countries and 76–85% in less-developed countries [1] [35]. However, it is not possible to directly compare such estimates with our study findings due to different criteria and methodologies used. Indeed, reliable data on mental health service utilisation and treatment gaps is extremely limited globally [35], and substantially more evidence on this is required, especially with conflict-affected populations.

Participation in the government’s general insurance scheme was positively associated with service utilization and especially GPs. However, despite this, costs related to services and drugs still represent major barrier for many. This finding is supported by other research conducted in Georgia (but not specifically on mental health) showing that the government’s general insurance scheme beneficiaries are more likely than non-beneficiaries to use general practitioners and specialist services [36] and pay less out-of-pocket payments for health services [37], [13]. However pharmaceuticals costs appear to have a high financial burden for both beneficiaries and non‐beneficiaries [13]. Costs related to drugs are main cost drivers and a cause of catastrophic health expenditure [38]. The other factor that may aggravate drug costs related barrier in mental health treatment is poor utilization of specialized mental health services. The patients enrolled in the SPMH are provided with the free medications. In our study every second of those who self-reported mental health problems but did not seek care mentioned costs related to medication as a barrier to access care. This might suggest that population is not well informed about benefits of the state program. The SPMH implemented by specialized outpatient mental health clinics (dispensaries) covers treatment of majority of mental health conditions including moderate and severe depressive episodes, recurrent depressive disorder and PTSD. Anxiety disorders such as phobic anxiety and other anxiety disorders are not included in the program coverage, meaning that the patient with these diagnoses should pay for consultation and purchase drug if needed. Medications provided by the state program are mainly low cost old generation drugs and generics. Only 2.3% of our study population with mental health disorders used outpatient mental health services and all of them received drug benefits from the program. Although the numbers are small it could indicate that psychiatric dispensaries are mainly visited for medications.

The majority of individuals with current symptoms of a mental disorder used pharmacy services and about one six used only a pharmacy without consulting a health professional. Such practice is referred as self-treatment because the pharmacist, if consulted, is not professional to prescribe medications for mental conditions. Self-treatment is common in the Georgian population [39] and it was found to be higher among uninsured persons as suggested by our study. Although the government’s general insurance scheme benefit package does not cover mental health drugs, extra costs related to service use for uninsured individuals is additional financial barrier prompting them to self-treatment. Self-treatment was supported by existing legal environment that did not restrict non-prescribed drug purchase at the pharmacies with exception of selected list of controlled narcotic drugs. Recent changes in the regulations imposed restrictions on majority of drugs, including those related to mental health.

Relatively high use of GP consultations (46.6%) may reflect the gate keeping role of primary care enforced by the government’s general insurance scheme. Also people with mental disorders may have other physical complaints that prompt them to seek care from GPs. Interestingly about one third used only the GP service without referring to other specialists. GPs should be able to recognise mental health disorders and manage mild depressive episodes, while referring more severe cases to psychiatrists. They are also authorised to prescribe antidepressants, however real quality of services with regards to mental health provided by GPs is not known and was not explored by our study.

As expected, neurologists at primary or secondary level are main access points for mental health treatment. They are equally consulted by insured and uninsured persons. The explanation could be that neurologists are main health care providers from which care is sought in case of mental and behavioural problems, although they have not been recognized as such in the policy decisions of the government. As pathways of treatment were not investigated we may assume that those who were insured were referred by GPs to neurologists, while uninsured most likely access neurologists directly bypassing general practitioners. However, this assumption needs further exploration and research.

High utilization of neurologist services and low utilization of specialized mental health services could be explained by stigma associated with seeking psychiatric care. Stigma as a major barrier to use psychiatric care has been documented by various studies [40], [41], [42]. In Georgia psychiatric outpatient clinics (dispensaries) are not integrated in the primary care, they are stand-alone facilities or attached to the psychiatric hospitals. Such model contributes to stigmatization of mental illness. Our study did not explore stigma and therefore this should be a subject for further research in Georgia.

The other factor that explains low use of specialized mental health services is poor quality of the government funded outpatient psychiatric care. A recent qualitative study that explored barriers in mental health care in Georgia identified poorly funded, low resourced outpatient psychiatric care as the most challenging among mental health services. There is low utilization of modern treatment modalities and existing funding models do not contribute to the quality improvement. There is no continuum of care and patients discharged from inpatient mental facilities are not followed-up. The staff is demotivated and overburdened. Due to absence of financial incentives the psychiatric field is not attractive to young doctors. [43]. A shortage of qualified staff is a recognized obstacle to mental health reform initiative in Georgia [12]. Psychosocial rehabilitation is provided by a few outpatient facilities under the SPMH, limited NGOs under the donor financial support and private clinics. The majority of respondents with current mental disorders reported receiving medication treatment, with very few receiving psychotherapy or psychosocial support, indicating possible over-medicalization. This reflects the limited coverage by additional services such as by NGOs and the unaffordability of costly private services.

In our sample about one third of those who screened for current mental disorder symptoms did not acknowledge having a problem requiring professional help. This possibly suggests poor mental health knowledge among the study population. There is growing evidence that poor mental health knowledge negatively influences decisions about mental health treatment [44], [45]. Other explanation could be self-reliance, which also is considered as barrier in not receiving care [46],[47]. On the other hand, not all mental disorders, especially mild conditions require treatment [48].

Utilization of services is affected by many interacting factors, such as individual and help-seeking preferences, access, availability of services and referral practices [49]. Health service utilization for mental health has not been studied in general population of Georgia. This once again underlines importance of our research as the study among war affected population may also provide some insight about utilization patterns in the general population in Georgia. No similar quantitative studies have been conducted in the neighbouring countries of Armenia and Azerbaijan.

The Global Burden of Disease (2010) study identified mental health disorders as a leading cause of burden. It is estimated that depressive disorders are second leading cause of years lived with disability in Eastern Europe [50]. To reduce this disease burden the government of Georgia should consider mental health as a public health priority and implement cost-effective interventions. A mental health reform has been recently initiated in Georgia. One of the directions and major challenges of the reform process is to integrate fragmented programs and services and close the treatment gap, including for war-affected populations in Georgia. However, in view of the magnitude of the problem, the government should make more proactive steps to meet the needs of people with mental disorders.

## Limitations

The study is subject to several limitations. The cross-sectional design precludes determining the direction of causation. In our study we present data on symptoms of mental health disorders rather than diagnosed mental disorders case. The period of such symptoms (within the past 1 or 2 weeks) differed from the period for the question on utilising health care for emotional and behavioural problems (1 year). It is possible that individuals may have remitted during last 12 months without treatment or due to successful treatment and such individuals would not classify for having current symptoms. In addition, the presence of symptoms of a mental disorder may not, in fact, indicate a need for care because those with mild conditions could remit without treatment. Thus we might have underestimated health service utilization rate in relation to real need. The wording of the question on seeking care for mental, behavioural or emotional problems during last 12 month could also lead to capturing those who actually did not have mental disorders. The study did not investigate participants’ experiences with health services, their satisfaction with received care, the quality of care, the pathways of care and the costs related to services and drugs. Another deficiency is that definitions of the counseling, psychotherapy and psychosocial support were not given which may have caused confusion in understanding. The study is also subject to recall bias as service use was measured for the last 12 months period. Selection biases should also be taken into consideration. IDPs hosted by relatives or friends or living independently away from the IDPs settlements were not included in the study. It is unclear whether this segment of IDPs have different service utilization pattern than those residing in collective centers. Another limitation was that we did not perform inter-rater reliability test for the data collectors. Lastly, the study instruments were not developed specifically for the study population and so may be prone to lack of cultural validity. However, they did go through a rigorous translation, adaption and piloting process, and the psychometric properties of the instruments were also tested and shown to be good (see above).

## Conclusions

The study suggests there is limited use of formal health services for mental health problems among war-affected population in Georgia with self-reported mental, emotional and behavioural problems and symptoms of mental disorders. This appears due to barriers such as costs of services and drugs. Reducing financial access barriers, especially for drugs, seems critical and the government in Georgia should consider expanding outpatient drug benefits and including the drugs needed for management of mental disorders. Another noteworthy finding of this study is that GPs and non-mental health specialists (neurologists) are the main service providers of “mental health services”. In contrast, specialised care is extremely underused and appears used only for free drug benefits. While many patients with mental health problems present to primary care, the real benefit to the patient is questionable unless the capabilities of primary health care are enhanced to deal with mental disorders. Integration of mental health into primary care with improved capacity of primary care providers, multidisciplinary treatment approach, and improved referral pathways could result in the more timely identification and successful management of mental disorders among war-affected persons in Georgia.
